# Development of a mucoadhesive drug delivery system and its interaction with gastric cells

**DOI:** 10.3762/bjnano.16.28

**Published:** 2025-03-13

**Authors:** Ahmet Baki Sahin, Serdar Karakurt, Deniz Sezlev Bilecen

**Affiliations:** 1 Department of Molecular Biology and Genetics, Konya Food and Agriculture University, 42080, Konya, Türkiye; 2 Molecular Biology, Genetics and Bioengineering Program, Faculty of Engineering and Natural Sciences, Sabanci University, 34596, İstanbul, Türkiye; 3 Department of Biochemistry, Faculty of Science, Selçuk University, 42130, Konya, Türkiye

**Keywords:** alginate, Eudragit RS100, mucoadhesive nanoparticles, mucus, smart drug delivery

## Abstract

Drugs that are designed for local treatment of gastric diseases require increased gastric residence time for prolonged action and increased efficacy. In this study, we report a mucoadhesive drug delivery system that was developed to fulfill these requirements. Alginate nanoparticles were synthesized by water-in-oil emulsification followed by external gelation and then coated with the mucoadhesive polymer Eudragit RS100. The formulated nanoparticles had a mean size of 219 nm and positive charge. A peptide, as a model drug, was loaded onto the nanoparticles with an encapsulation efficiency of 58%. The release of the model drug from the delivery system was pH-independent and lasted for 7 days. The periodic acid–Schiff stain assay indicated 69% mucin interaction for the nanoparticles, which were also capable of diffusion through artificial mucus. The nanoparticles were not toxic to gastric epithelial cells and can be internalized by the cells within 4 h. The adsorption of nanoparticles onto mucus-secreting gastric cells was found to be correlated with cell number. The delivery system developed in this study is intended to be loaded with active therapeutic agents and has the potential to be used as an alternative drug delivery strategy for the treatment of gastric related diseases.

## Introduction

Drug delivery through the oral route is the most preferred route because of ease of application, high patient compliance, and non-invasiveness [1]. Drug formulations designed for the oral route are aimed for two delivery approaches, namely, (i) systemic drug delivery, in which the drug must be absorbed by the mucosa into the systemic circulation, and (ii) local delivery to treat local diseases such as gastric and colorectal cancers or local bacterial infections. In this case, the drug will not be systemically absorbed into the circulation, but it will become effective at the local site [2]. For both situations, the effectiveness of the drug formulation depends on several factors such as gastric residence time, gastric emptying, release rate of the drug from the dosage form, and the therapeutic agent reaching the site of action or absorption. Conventional drug delivery systems may not be effective in overcoming these obstacles, particularly regarding drugs that are designed for the treatment of local gastric diseases [3]. The solution to this is the development of gastroretentive drug delivery systems. These are designed to increase the drug residence time in the upper part of the gastrointestinal system, which leads to higher effectiveness [4–5]. Over the years, different types of gastroretentive drug delivery systems have been developed, including floating, expandable, high-density, or mucoadhesive systems [6]. Among these, mucoadhesive systems are quite effective in localizing the drugs to the site of action because of increased drug retention at the mucosa [7]. These systems have the capacity to strongly adhere to the mucus layer, provide slow release of its contents, and even reduce the required dose because of higher accumulation of the drug at the target site [8].

Nanoparticles synthesized from mucoadhesive polymers such as chitosan, alginate, cellulose, polyacrylic acid, and polymethacrylic acid have been introduced as gastroretentive drug delivery systems. The mucoadhesive properties of these polymers are attributed to electrostatic bonding between polymer and sialic acid of mucin, hydrogen bonding, disulfide bond formation, or physical entanglement of polymers within the mesh-like mucus structure [9–10]. Sodium alginate is a linear polysaccharide composed of 1,4-linked β-ᴅ-mannuronic acid and α-ʟ-guluronic acid residues. Alginate can be used to form porous matrix-type drug delivery systems because of their ability to form gel-like structures in the presence of divalent cations such as Ca^2+^. Despite the advantages of alginate polymer such as its biodegradability, biocompatibility, and gelation ability, its instability, fast wettability, and rapid release at high pH result in the leakage of encapsulated drugs. These drawbacks make alginate challenging to be used in drug delivery applications [11–12]. Therefore, it is generally used together with other polymers, such as chitosan [13] or carboxymethyl cellulose [14], or it is modified with PEG-maleimide [15] to acquire mucoadhesion, effective encapsulation, pH-responsive release, and enhanced stability.

Eudragit RS 100 is a copolymer of ethyl acrylate, methyl methacrylate, and a low proportion of methacrylic acid ester with quaternary ammonium groups. It is known for its mucoadhesive properties [16], which are independent of the site of the mucoid surface [17]. Eudragit RS100 polymer has been used for several applications aimed at different sites of the body such as skin [18], intestinal [16], intranasal [19], or ocular [20] drug delivery. This broad range of application sites is possible because pH-independent swelling of the polymer enables drug release by diffusion [21]. Eudragit RS30D is the 30% aqueous dispersion of Eudragit RS100, which is promptly used as coating material [22] or within formulations of drug delivery systems with sustained release characteristics [23]. Although the mucoadhesion of this polymer is known, there are few studies focusing on this property when they are used as nanoparticle formulations [17].

Over the years, several valuable alginate-based applications have been reported as gastroretentive drug delivery systems, in which alginate beads were either coated with aminated chitosan [24], or alginate was blended with a plant-based polysaccharide [12] or used together with carboxymethylcellulose [25] to improve the mucoadhesion of the designs. To our knowledge, there are not many studies that use the properties of alginate and Eudragit RS100 polymer in a mucoadhesive gastroretentive delivery system to be used in stomach delivery applications. In one study, alginate and various mucoadhesive polymers, including Eudragit RS100, were blended to deliver an anti-ulcerative drug to the stomach for prolonged gastric retention. Approximately 67% of an Eudragit microsphere formulation was found to adhere to the mucosa. The size of the microspheres, from which the drug was released over a period of 24 h, was in the range of 800–900 µm [26]. Although particulate systems with larger sizes could be advantageous in terms of higher encapsulation efficiency and slower release, they would have a reduced surface area for adhesion. Also, mucus penetration would be hindered because of the mesh-like structure of mucin. For therapeutics that have gastric mucosa as target, this might limit the efficiency and decrease the drug absorption at the site. A smaller particle size, however, is advantageous because of the larger surface-area-to-volume ratio, which may result in more contact points with the tissue and increased mucoadhesion [27]. In addition, a nanoscale size leads to improved penetration through the pores of the mucin network, which have a size of approximately 500 nm [9,28], enhanced retention within the mucus because of stronger interaction [29], and better and uniform distribution throughout the gastric mucosa [2].

The motivation behind the current study, therefore, was to synthesize a nanoscale drug delivery system with mucoadhesive properties in an attempt to achieve improved gastric action of drugs with increased retention. We have developed an mucoadhesive alginate-based drug delivery system to be used in the stomach delivery of drugs. For the study, we used a carboxyfluorescein (FAM)-labeled peptide (*M*_w_ = 2.8 kDa) as a model drug to be encapsulated in alginate nanoparticles. The negatively charged alginate nanoparticles were then coated with positively charged Eudragit RS100 with the intent of obtaining mucoadhesive nanoparticles with sustained release properties. The characterization of the delivery system was studied in terms of charge, size, encapsulation efficiency, and release rate. The mucoadhesive characteristic of the system was tested in situ and in vitro. In addition, the in vitro cytotoxicity and internalization of the nanoparticles by mucus-secreting gastric cells were also investigated. We believe that usage of the developed system may increase the retention time of the drugs within the mucus microenvironment of the stomach and, thus, may lead to elevated local activity or absorption of the therapeutic agents from the mucosa.

## Results and Discussion

### Morphology of nanoparticles

**Figure 1:**
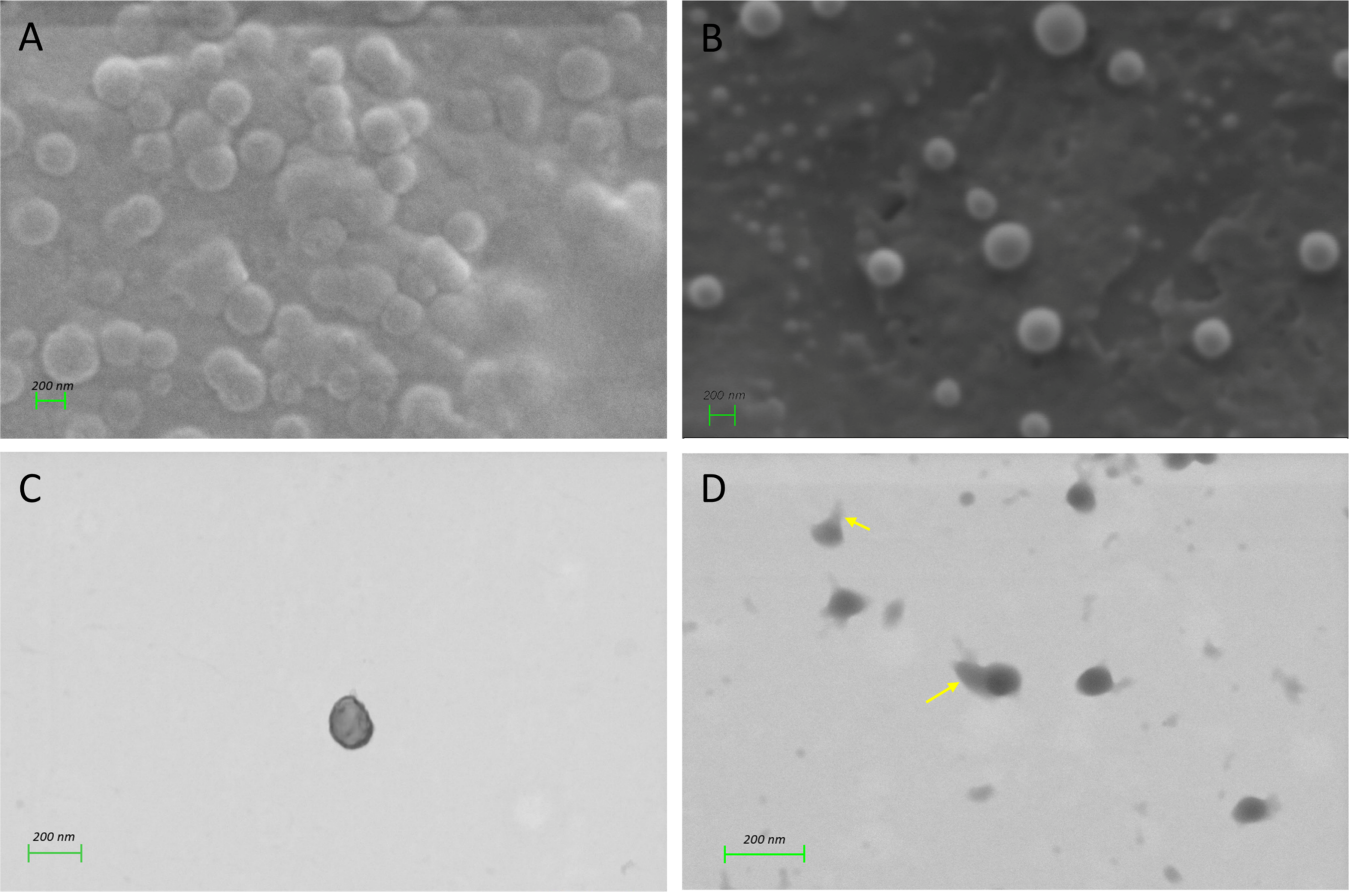
Micrographs of nanoparticles. SEM micrographs of (A) Alg NPs and (B) EudAlg NPs. STEM micrographs of (C) Alg NPs and (D) EudAlg NPs. Scale bars: 200 nm.

After the synthesis of alginate (Alg) and Eudragit-coated alginate (EudAlg) nanoparticles, topography, surface composition, size, and charge distribution of the delivery system were determined. The topography of the nanoparticles was studied with SEM (Figure 1). Both Alg and EudAlg nanoparticles are spherical with smooth surfaces (Figure 1A,B). It should be noted that during SEM evaluation the electrons send onto the specimen scan the surface, and the signal collection involves collecting secondary electrons. Hence, it is difficult to distinguish surface modifications of the nanoparticles and to separate the core of the nanoparticles from the shell. Therefore, the Eudragit coating was characterized by STEM, where transmitted electrons are used to create the image [30]. In STEM micrographs, alginate nanoparticles appeared with sharp edges; however, the edges of the EudAlg nanoparticles revealed secondary projections (Figure 1C,D). Similar micrographs in which the edge of the nanoparticles appeared as secondary coating or projections when modified with polymers were observed in literature [31–32]. Therefore, the secondary projections, shown in yellow arrows in Figure 1D, around the nanoparticles are attributed to the presence of Eudragit RS100 polymer around the Alg nanoparticles.

In addition to morphological characterization, the coating of Alg nanoparticles with Eudragit RS100 polymer was studied by using fluorescently tagged polymers during particle synthesis (Supporting Information File 1). For the preparation of fluorescently tagged Alg (F-Alg) nanoparticles, fluorescein isothiocyanate (FITC)-tagged alginate polymer was purchased and used (RuixiBiotech, China). Eudragit polymer was labeled with rhodamine B isothiocyanate (Rh-Eud polymer) as mentioned in Supporting Information File 1. The F-Alg nanoparticles were then coated with Rh-Eud polymer resulting in Rh-Eud-F-Alg NPs. To determine if the F-Alg nanoparticles were coated with Rh-Eud polymer, the fluorescence spectra of F-Alg NPs, Rh-Eud-F-Alg NPs, and Rh-Eud suspension, which did not contain F-Alg NPs, were obtained. The nanoparticles were excited at the excitation wavelength of F-Alg NPs (λ_ex_ = 490 nm), and the emission spectra of the samples were scanned between 515 and 595 nm. F-Alg nanoparticles revealed maximum emission at 530 nm, while the Rh-Eud suspension without F-Alg NPs did not show emission. However, when Rh-Eud-F-Alg NPs were excited at 490 nm, peaks were observed at both 530 and 575 nm (Supporting Information File 1, Figure S1). This observed secondary peak in Rh-Eud-F-Alg NPs may indicate the close proximity between Alg NPs and Eudragit polymer, that is, upon excitation at 490 nm, Alg nanoparticles emit fluorescence at 530 nm, and this emission can excite rhodamine B and cause emission at 575 nm in Rh-Eud-F-Alg NPs.

### Surface composition of nanoparticles

**Figure 2:**
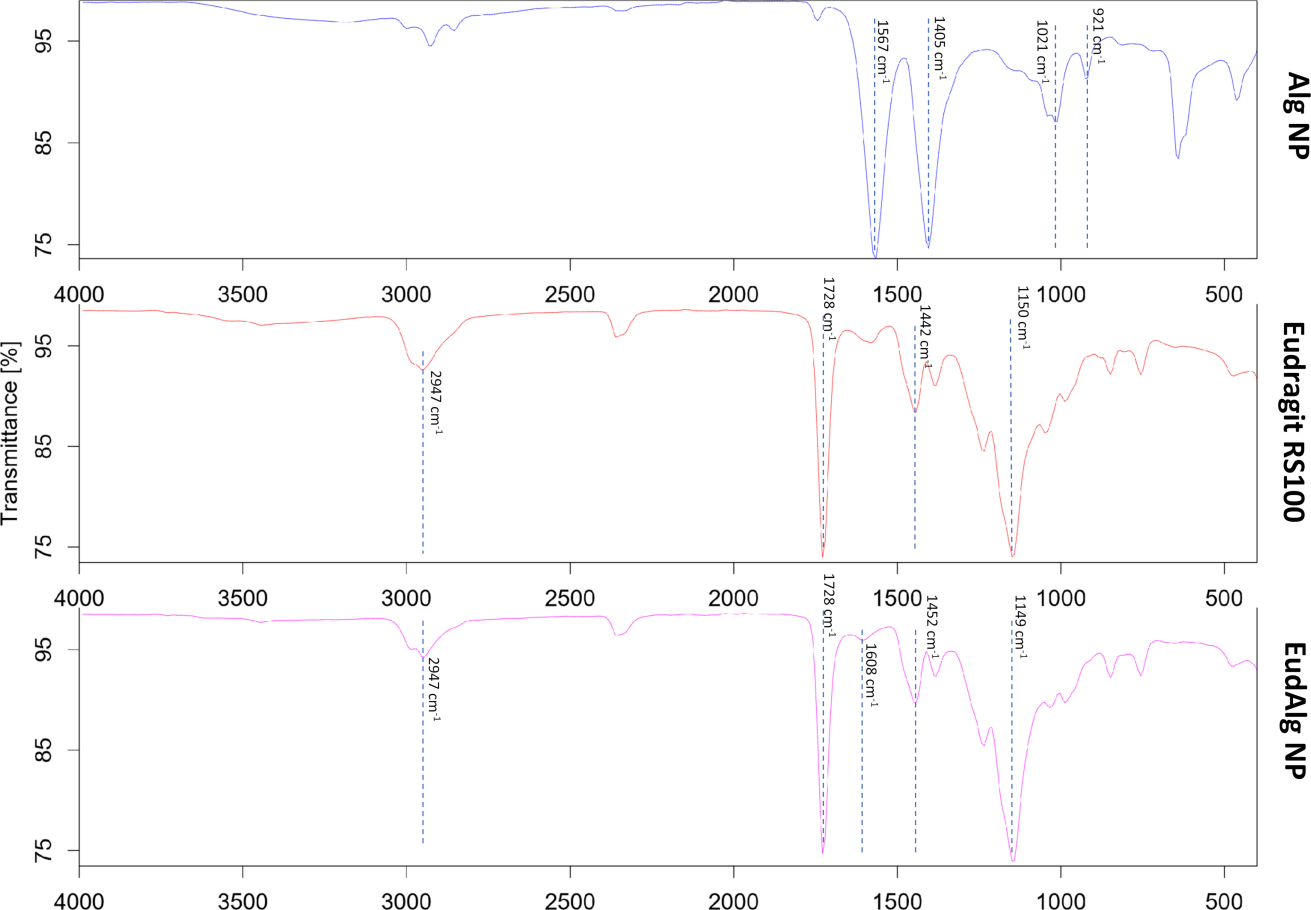
ATR-FTIR spectrum of Alg NPs, Eudragit RS100 polymer, and EudAlg NPs.

The surface composition of the nanoparticles was characterized with ATR-FTIR (Figure 2). For Alg nanoparticles, distinctive bands were observed at 1567 and 1405 cm^−1^, which were assigned to asymmetric and symmetric stretching vibrations of carboxylate groups [33]. Other peaks corresponded to C–C stretching vibrations of pyranose rings (at 1021 cm^−1^) and C–O stretching vibrations of uronic acid residues (at 921 cm^−1^) [34]. For Eudragit RS30D polymer, the peak at 2947 cm^−1^ represents C–H stretching, and the peak at 1150 cm^−1^ corresponds to ester groups. The peaks at 1728 and 1442 cm^−1^ refer to ester C=O stretching vibrations [35] and –CH_3_ bending of alkanes, respectively [36]. When the spectrum of EudAlg nanoparticles was analyzed, the characteristic peaks of Eudragit polymer could be observed. In addition, a slight shift in alginate COO^−^ peak position was detected (from 1567 to 1608 cm^−1^). This was attributed to weak interactions between alginate and Eudragit polymer, which were also observed in other studies [37]. Taken together, it can be concluded from the results of microscopy and FTIR analysis that the Alg nanoparticles were coated with Eudragit polymer.

### Particle size and zeta potential distribution

The particle size distribution is an important parameter in drug delivery applications because it determines the transport across membranes. The Z-average diameters of Alg NPs and EudAlg NPs were 206.14 ± 32.31 and 219.22 ± 41.61 nm, respectively (Table 1). Eudragit RS100 polymer in suspension had a size of 192.74 ± 11.13 nm, which was also observed in other studies conducted with this polymer [20,38].

**Table 1:** Z-average, polydispersity index (PDI), and zeta potential of the nanoparticles and Eudragit RS100 suspension.

	Z-average (nm)	PDI	Zeta potential (mV)

Alg NPs	206.14 ± 32.31	0.23	−25.85 ± 7.7
EudAlg NPs	219.05 ± 41.61	0.24	39.72 ± 6.7
Eudragit RS100 suspension	192.74 ± 11.13	0.14	44.42 ± 8.9

There is a wide range of alginate particles sizes (100 to 1000 nm) reported in the literature [39–40], which change based on the method used for nanoparticle preparation. The nanoscale size is particularly important in mucoadhesive systems designed for gastric delivery because of the mesh-like structure of gastric mucus. Since the pore size in gastric mucus is around 500 nm [9], the smaller the nanoparticle, the better the mucus diffusion [41]. Larger particles may be filtered out, which reduces the absorption of therapeutic agents from the mucosa. Specifically, for this study, obtaining nanoscale Alg nanoparticles was critical since these particles were to be coated with a second polymer, Eudragit RS100, which would lead to increase in size and impair the mucus interaction. According to the particle size results, the coating did not significantly increase the size of nanoparticles (*p* > 0.05). Even after coating, the final EudAlg NPs were still in the nanometer range.

For efficient mucus interaction, the charge of the particles is also very important. The zeta potential of Alg nanoparticles is negative (−25.85 ± 7.7 mV), as expected, because of the presence of –COOH and –OH groups in the polymer. This may hinder its interaction with negatively charged surfaces like mucus because of charge repulsion [42]; in contrast, positively charged nanoparticles have more potential to induce adherence to the mucus layer [43]. Thus, for this study, Eudragit RS100 polymer was chosen as a coating polymer to obtain positively charged nanoparticles. Upon coating the Alg nanoparticles, the zeta potential shifted to positive values (39.72 ± 6.7 mV), which is another indicator of successful coating (Table 1). From these results, it can be deduced that the synthesized cationic nanoparticles, which are smaller than the pores of gastric mucus, may have the advantage of adherence and diffusion to the gastric mucus.

### Encapsulation efficiency of EudAlg nanoparticles

Encapsulation efficiency is an important parameter in determining the dose of therapeutic agents and, thus, the efficacy of the treatment. In the present study, we aimed to determine the encapsulation of a positively charged and large peptide molecule (*M*_w_ = 2.8 kDa) into the delivery system. The reason for choosing this peptide as a model drug was that there is ongoing research for the use of peptide-based therapeutics for the treatment of diseases; however, development of oral peptide therapy is quite challenging because of the high acidity in the stomach and the presence of the protein-digesting enzyme pepsin [44]. Despite the challenges, remarkable developments are unfolding. Recently a peptide-based therapeutic delivery system that is absorbed in the stomach was approved by the FDA as oral formulation to be used in type-II diabetic patients [45]. This inspired us to develop and test systems for the delivery of peptide-based therapeutics. The peptide used in this study was synthesized with the fluorescent label 5-carboxyfluorescein (5-FAM) at the N-terminal and was purchased from Pepmic, China. The fluorescent label on the peptide was introduced to ease tracing the peptide within the nanoparticles by using fluorescence measurements. The properties and the emission spectrum of the peptide are provided in Supporting Information File 2. For the calculation of encapsulation efficiency, a calibration curve with the known concentrations of labeled peptide and the corresponding fluorescence intensity (FI) values (λ_ex_ = 485 nm, λ_em_ = 530 nm) was used to calculate the amount of peptide within the nanoparticles. Based on the parameters used for the nanoparticle synthesis in this study, the encapsulation efficiency of the FAM-labeled peptide model within EudAlg NPs was (58.6 ± 3.9)%. To our knowledge, the range of encapsulation efficiency (EE) values determined in studies conducted with alginate-based nanoparticles was 7–90% [46–47]. As examples, Fernando et al., produced nanoparticles by a water-in-oil emulsification/external gelation process with EE values of 36% [48]. Sadeghi et al. prepared alginate microparticles with EE values of 73% and 69% with two different model drugs [49]. Alizadeh et al. synthesized alginate-based nanocarriers with 68.4% encapsulation efficiency [50]. The encapsulation efficiency value obtained in this study was found to be promising and comparable with other studies in the literature. However, it might be considered relatively low, the reason of which might be the high amount of model drug used as an input during nanoparticle preparation (which determines the EE%). It should be noted, however, that the potency of the drug itself is the other parameter of crucial importance when considering the efficiency of a drug delivery system. Therefore, further optimization studies may be required to improve the encapsulation efficiency of the nanoparticles, if the therapeutic agent to be used requires higher loading values to be effective.

### Release from EudAlg nanoparticles

**Figure 3:**
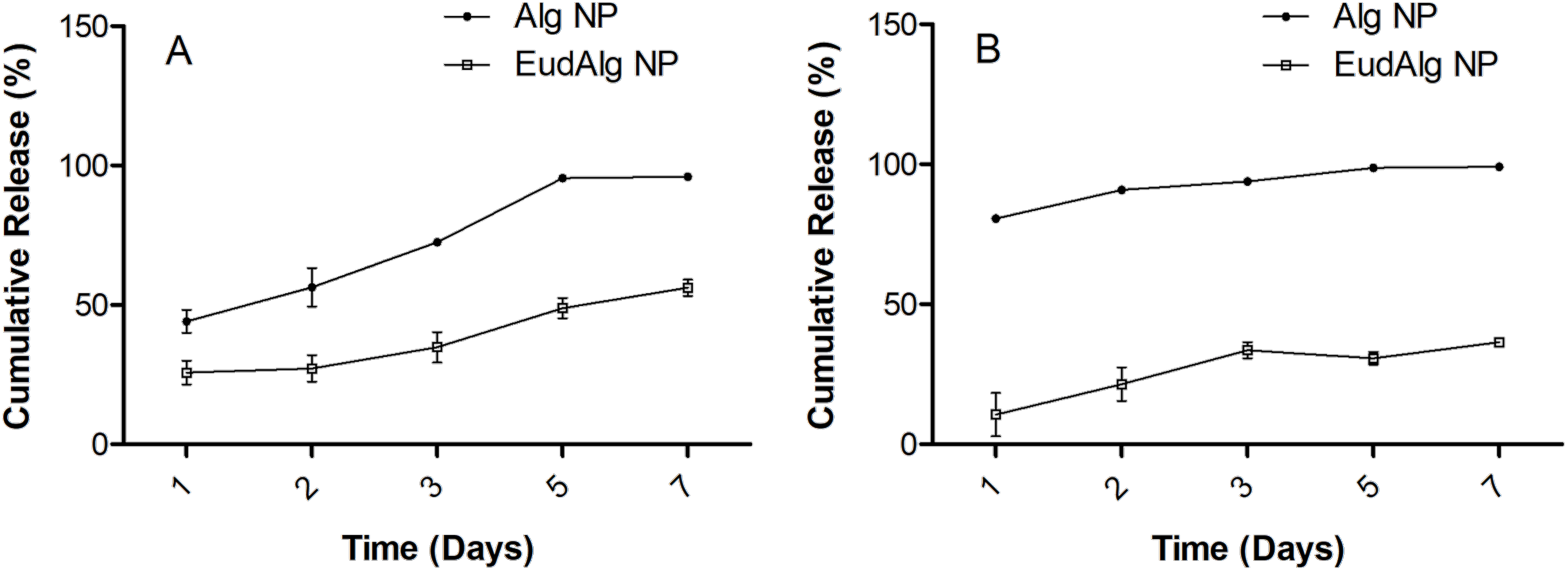
Cumulative release of FAM-labeled peptide. (A) At pH 1.2 and (B) at pH 6.8.

The purpose of the present study was to develop a system to deliver the therapeutic agent and make it available locally at the gastric mucus. One important parameter is the release pattern of the drug from the formulation. Therefore, to study the release, the nanoparticles loaded with FAM-labeled peptide were suspended in different dissolution media (pH 1.2 and pH 6.8) and incubated for 7 days (Figure 3). At specified time intervals, the nanoparticles were centrifuged and resuspended in dissolution medium, and the fluorescence of the resuspended nanoparticles was measured. The purpose of the different dissolution media was to investigate possible release differences under different pH conditions. It is known that gastric mucus is acidic (around pH 2) at the luminal site and almost neutral (around 6) at the epithelial surface [9]. Since the drug delivery system is expected to adhere to the mucus layer and diffuse into it, it is important to determine the release differences, if there are any, at this pH gradient. According to the results of the study, the presence of Eudragit polymer on alginate nanoparticles slows down the release at both pH values. Alg nanoparticles release their content much faster at neutral pH, when compared to the acidic pH. This difference, as also observed by others, can be explained by a smaller degree of ionization of alginate polymer at acidic pH, resulting in the maintenance of its structure and content [51]. From this result, it can be concluded that the presence of Eudragit polymer on the alginate nanoparticles as a coating is beneficial, particularly at neutral pH because after the nanoparticles reach the mucus and start diffusing, the coating would protect and slow down the otherwise much faster release of the drug to the site.

### In situ mucoadhesion studies

The ability of nanoparticles to stick to mucus, known as mucoadhesion, is important for retention at mucus-bearing sites and, thus, prolonged drug release into the environment [52]. The mucus interaction of the EudAlg NPs was first studied by DLS measurements, where the particle size and zeta potential distributions were recorded upon interaction with mucin. Table 2 shows the results of the DLS measurements. After EudAlg nanoparticles came in contact with mucin, the Z-average value of the nanoparticles increased significantly from 219.05 ± 41.61 nm to 387.08 ± 121.8 nm. The increase in size after mucin interaction was observed in other studies as well [52–54]. The size increase of the particles interacting with mucin has been attributed to the formation of bigger aggregates or complexes due to the interactions of positively charged groups on the nanoparticles and negative charges on mucin. The increased overall size distribution due to formation of bigger aggregates can also be observed by analyzing the polydispersity index (PDI) values obtained from the DLS measurements. The PDI indicates the homogeneity of the size of the nanoparticle suspension, and it is stated that the higher the increase in the PDI value of the nanoparticle suspension, the better the interaction of the particle with mucin. The PDI values of EudAlg nanoparticles increased from 0.24 ± 0.08 to 0.39 ± 0.15 after the interaction with mucin, suggesting the formation of bigger complexes after mucin interaction.

**Table 2:** DLS measurements of nanoparticles before and after mucin interaction.

	Before mucin interaction	After mucin interaction

Z-average (nm)	219.05 ± 41.61	387.08 ± 121.8
PDI	0.24 ± 0.08	0.39 ± 0.15
zeta potential (mV)	39.72 ± 6.7	−12.13 ± 9.8

In addition to the size distribution and PDI alterations, the zeta potential of EudAlg nanoparticles shifted from 39.72 ± 6.7 to −12.13 ± 9.8 mV after interaction with mucin. The observed reduction in zeta potential is also attributed to the interaction and surrounding of EudAlg nanoparticles with mucin, which is negatively charged because of sialic acid residues [53,55]. The change in size and zeta potential after incubation with mucin indicates that EudAlg nanoparticles interact with mucin [56]. The interaction of mucoadhesive polymers with mucin occurs through intermolecular hydrogen bonding and secondary interactions resulting from the interpenetration of polymer chains of mucin into the structure of the nanoparticles. The presence of positively charged Eudragit polymer on the surface of nanoparticles yield additional electrostatic interactions, which are known to be one of the driving force behind mucoadhesion [57]. Therefore, the presence of the Eudragit polymer layer on the designed nanoparticles is beneficial for the enhancement of the electrostatic interactions between the nanoparticles and the gastric mucus, thus increasing their entrapment into the mucus layer.

### Periodic acid–Schiff stain assay

The periodic acid–Schiff (PAS) stain assay quantifies the degree of interaction between nanoparticles and mucin. To this purpose, the particles were incubated with mucin for different time intervals and then centrifuged. The supernatant representing the unadsorbed mucin was measured, and the data obtained from the study are presented as percent adsorbed mucin onto the nanoparticles with respect to the reference (where only mucin was used). The results of the study indicated that (62 ± 5)% of mucin was adsorbed onto the EudAlg NPs within 15 min of incubation; after 1 h this value increased to (69 ± 1)%, which is not statistically significant. This suggests that the surface of the nanoparticle is saturated with mucin within the first 15 min because of the formation of weak interactions such as hydrogen bonds, van der Waals forces, and ionic interactions. Although a direct correlation with nanoparticles of different surface chemistries is challenging, comparable results were reported with mucoadhesive systems in particulate forms [58–59]. The results of the PAS stain assay together with the changes in the size and zeta potential values after mucin interaction of the EudAlg nanoparticles reveal that the synthesized EudAlg nanoparticles have mucoadhesive properties.

### Interaction of nanoparticles with artificial mucus as a simple model

Prolonged residence time in gastric mucosa is important for reduced elimination of particulate systems from the stomach. Mucoadhesive drug delivery systems eventually lead to an increased amount of drug at the mucosa. However, these nanocarriers might also be trapped in the mucus layer and get washed away with mucus turnover. The ability to penetrate through the mucus layer and to reach the underlying epithelium are therefore important parameters to be considered when developing mucus-interactive drug delivery systems. To study the possible penetration of the designed nanoparticles through the mucus layer, artificial mucus was prepared using gastric mucin, which was placed on a hardened gelatin layer. The penetration of nanoparticles was examined after 24 h through fluorescence measurements in the underlying gelatin layer (test group). As the reference group, nanoparticles were dispersed in water (instead of mucin) and incubated on a gelatin layer for 24 h. The purpose of this group was to determine the maximum nanoparticle diffusion into the gelatin layer due to other characteristics of the nanoparticles such as nanoscale size and charge [60]. The results of the study revealed a moderate diffusion of (31 ± 3)% in the test group with respect to the reference group. There are studies in literature that took a similar approach to test the penetration of mucoadhesive nanoparticulate systems. For instance, Ungaro and colleagues designed poly(lactic-*co*-glycolic acid) nanoparticles with different modifiers and revealed 10% diffusion into the gelatin layer. In addition, their study revealed the effect of particle charge on the ultimate diffusion through the mucus; positively charged particles exhibited higher diffusion than negatively charged particles [61]. Ünal et al. studied drug-loaded pristine and polyethylenimine- or chitosan-coated cyclodextrin-based nanoparticle formulations and measured drug mucus penetration percentages ranging from 45% to 73% [62]. They concluded that this difference was not only due to the charge of the nanoparticles but also due to the different sizes of the formulations. In a recent study, the effects of surface charge and chemistry of the nanoparticles on their penetration abilities were assessed with a similar approach in a newly designed synthetic mucus hydrogel barrier system. The authors used differently sized polystyrene nanoparticles with polyethylene glycol (PEG) modifications. PEGylated nanoparticles exhibited better penetration than non-PEGylated formulations, regardless of the particle size. However, the penetration of PEGylated formulations of different sizes was also studied, and a negative correlation was observed with particle size. In the same study, the effect of gel thickness was evaluated, and it was concluded that the penetration was negatively correlated with mucus gel thickness. Regardless of the particle size, penetration was found to be significantly lower when a thicker gel layer was used [63]. In summary, not only charge but also the size of nanoparticles, along with the mucus thickness, affect the diffusion of nanoparticles. The relatively low diffusion percentage obtained in our study here fits into the diffusion rates obtained in the literature. The moderate diffusion might be additionally explained by the entrapment of nanoparticles into the mucus, either through their mucoadhesion capability, or through the collapse of mucus fibers into bundles around the nanoparticles, leading to immobilization of the carriers [64]. It should also be noted that the mucus layer through which the nanoparticles must diffuse to reach to the underlying gelatin layer (where the fluorescence measurements were taken from) is approximately 7 mm thick. The mean mucus thickness in stomach is 190–275 µm [65], with about 150 µm of this layer being firmly adherent over the mucosal surface in the antrum [66], and the mucin turnover in stomach is around 10 h [67]. Judging from this information, the diffusion of nanoparticles through a mucus layer of 7 mm thickness in 24 h, may actually be a promising result when considering in vivo conditions. Therefore, further studies such as multiple particle tracking, with which penetration distances on the micrometer scale over seconds can be determined, might be required to clarify the penetration capability of the particles through the mucus. In this way, the possible mucus penetration in the stomach may be deduced more accurately.

### Determination of cell viability

**Figure 4:**
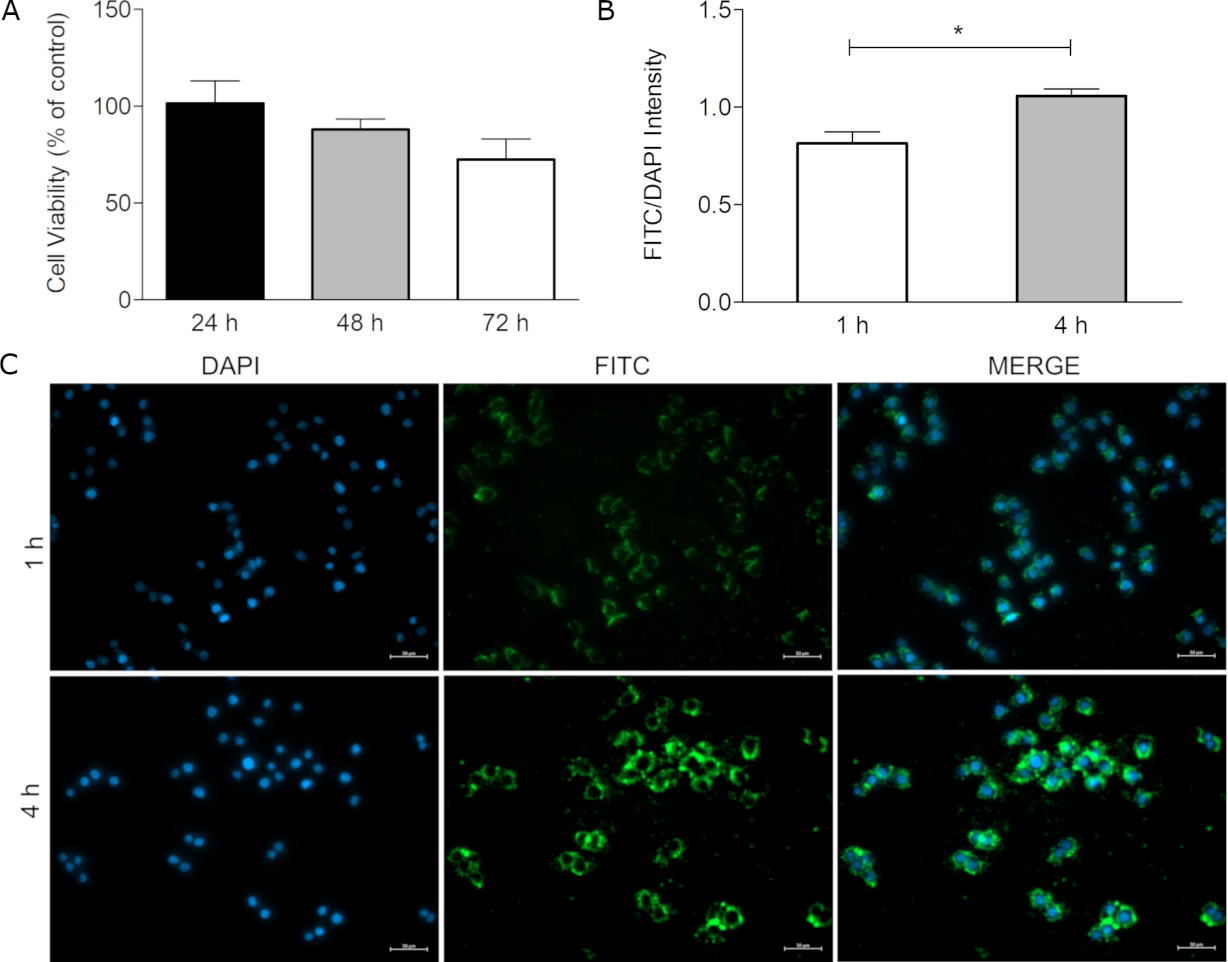
Viability and internalization of nanoparticles into AGS cells. (A) Viability (%) of AGS cells treated with nanoparticles, (B) FITC/DAPI intensity ratios of AGS cells after internalization of nanoparticles for 1 and 4 h, and (C) representative micrographs of nanoparticle internalization after 1 and 4 h.

Possible toxic effects of EudAlg NPs on the human gastric adenocarcinoma cell line AGS were assessed for three days in 24-well plates for 1 × 10^5^ cells and 1 mg/mL NPs per well. The cells were seeded, incubated for one day for complete attachment, and then treated with nanoparticles for 3 days. The data is presented as percent viability, which is the proportion of metabolic activities of treatment and control group. On day 1, the viability of the EudAlg NP-treated group was found to be (101.6 ± 11)%, indicating a viability similar to that of the control group. On the following two days, the viability values were (88 ± 5)% and (72.6 ± 11)%, respectively. No statistical significance was observed for the cellular viability after three days. Based on this result, it was concluded that EudAlg nanoparticles do not significantly affect the viability of AGS cell (Figure 4A).

### Internalization of nanoparticles

When nanoparticles are designed for biomedical applications, two important properties to be considered are toxicity and cellular uptake. The cellular uptake of nanoparticles is a dynamic process where both endocytosis and exocytosis are involved. The uptake also depends on the concentration of nanoparticles and the duration of the process. Studies conducted with the AGS cell line revealed that nanoparticle internalization generally reaches a plateau within the first 2 h [68–69]. In other studies performed with other cell lines with various nanoparticles, the progression in internalization was generally followed for 3 or 4 h or directly checked 4 h after treatment [70–72]. Therefore, for this study, the progressive internalization of EudAlg NPs was studied after 1 and 4 h of treatment to determine the efficiency of acute cellular uptake of nanoparticles. Mucus-secreting AGS cells were incubated with fluorescently labeled EudAlg (F-EudAlg) nanoparticles to track the internalization. The cell nuclei were stained with DAPI, and micrographs were taken with a fluorescence microscope (ZEISS, Axio Observer 3) (Figure 4C). The green signal obtained from F-EudAlg nanoparticles increases with incubation time. To determine the increase in internalization in a more quantitative way, the FITC and DAPI intensity values were obtained from eight different images by using the ZEISS ZEN software. A significant increase of the ratio between the intensities of FITC and DAPI was observed from 1 to 4 h. This suggests that the nanoparticles can be internalized without damaging the cultured cells within 4 h. They can cross the cell membrane and accumulate in the cytoplasmic region in a time-dependent manner.

### In vitro mucus interaction studies

To analyze the interactions of nanoparticles with mucins in vitro, mucus-secreting AGS cells [69] were used. The mucus secretion of cells under our culturing conditions were determined via the PAS stain assay. The evaluation of mucin secretion from AGS cells was studied after two days of culturing on 24-well plates, and purple-red-stained cells were considered as mucin-positive cells [73] (Supporting Information File 3). AGS cells are known to express both secreted mucins (such as MUC5AC) and membrane-bound mucin (MUC1) [74–75]. The interaction of nanoparticles with mucus-secreting AGS cells was investigated by using two different cell concentrations. The purpose of this experiment was to investigate whether the increased cell number and the increased mucin presence on the cells would correlate with nanoparticle adherence onto the cells. For the experiment, two control groups were used. The first group was wells containing only the cells, to which no nanoparticles were administered. The purpose of this group was to eliminate any false fluorescence that may be emitted from the cell pellet. The second control group was empty wells with nanoparticle administration, the purpose of which was to eliminate non-specific adsorption of nanoparticles onto the well. The calculation of relative mucus interaction was performed as described elsewhere [76] with the formula given in the Experimental section. Based on the calculations, the fractions of interaction were 4.5 ± 2.7 and 7.4 ± 0.3 for 6 × 10^4^ and 1.2 × 10^5^ cells/well, respectively. As expected, the nanoparticle adsorption onto the cells depends on the cell concentration. Since the fluorescence measurements were carried out on the cell pellets after washing and centrifugation, the increase in obtained fluorescence represents the increased amount of nanoparticles interacting with the cell membrane and might be associated with the increased mucin amount along with the cells. This indicates that the nanoparticles can be adsorbed onto the cells under in vitro conditions.

## Conclusion

Gastric targeting of drugs requires increased gastric retention to elevate the amount of drug at the site of action. In this study, a mucoadhesive drug delivery system was developed. The fabricated nanoscale particulate system reveals positive charge, which is beneficial for mucus interaction. The entrapment efficiency of the model peptide drug was 58%, the release of which was slowed down by the presence of Eudragit polymer on the nanoparticles. The nanoparticles indicated promising mucoadhesive properties as shown by DLS measurements and PAS stain assays. The delivery system had moderate diffusion capability through artificial mucus. The nanoparticles were internalized by mucus-secreting AGS cells within four hours, while exhibiting no toxicity towards the cells. The sufficient mucoadhesive properties, biosafety, and internalization into the cells indicate the potential use of the delivery system as oral drug carrier towards gastric sites. By loading the nanoparticles with different drugs proposed for several gastric diseases such as gastric ulcers, gastritis, bacterial infections, or cancer, the efficacy of treatments for the diseases might be elevated.

## Experimental

### Materials

Sodium alginate (medium viscosity, A2033), poloxamer 407 (16758), calcium chloride, and mucin from porcine stomach (M1778) were purchased from Sigma-Aldrich, USA. Sunflower oil and sorbitan monooleate (Span 80) were from TCI Chemicals, USA. For in vitro studies, high-glucose DMEM and Pen-Step solution were purchased from Sartorius, Germany. Fetal bovine serum was from Biological Industries, USA, and alamarBlue cell viability reagent was purchased from Invitrogen. For microscopy studies, PureBlu DAPI was purchased from BIORAD, and FITC-alginate was purchased from RuixiBiotech, China.

### Preparation of alginate nanoparticles

Alginate nanoparticles (Alg NPs) were prepared with a technique similar to the one used in [77]. Briefly, sodium alginate (1.35 mL of 0.5% w/v) and sunflower oil (6.75 g) containing Span 80 (405 µL) were emulsified at 30000 rpm for 5 min. The emulsion was then probe-sonicated in an ice bath (50 s pulses on and 10 s off, 40% Amp) for 10 min. At the end of sonication, CaCl_2_ (0.22 M, 1 mL) containing poloxamer 407 (1%, w/v) was dripped with an injector (Genject, Türkiye) into the mixing emulsion with a syringe pump at a rate of 6 mL/h. The final emulsion was probe-sonicated in an ice bath (50 s pulses on and 10 s off, 40% Amp) for 5 min, followed by 30 min of incubation on a magnetic stirrer. Finally, isopropanol (4 mL) was added to the emulsion, and the nanoparticles were collected by centrifugation (3000*g* for 5 min). The pellet was washed once with isopropanol and three times with double-distilled water (ddH_2_O). The nanoparticles were resuspended in sodium acetate buffer (pH 4.5) and sonicated for 5 min in a sonication bath before characterization.

### Preparation of Eudragit RS100-coated alginate NPs

The Alg NPs were coated with Eudragit RS100 by a technique similar to that in [20]. Prepared Alg NPs (1 mg/mL; 4 mL) were dripped into Eudragit RS 100 suspension (2%, v/v, of Eudragit RS 30D, diluted with ddH_2_O) by using a syringe pump at a rate of 5 mL/h, and the dispersion was incubated on a magnetic stirrer (700 rpm, 10 min, room temperature). At the end of the incubation, coated NPs were centrifuged (10000*g* for 10 min), washed once with ddH_2_O, and sonicated for 5 min in a sonication bath before characterization.

To investigate the interaction of EudAlg nanoparticles with artificial mucus and for visualization of nanoparticles in fluorescence microscopy, fluorescently labeled Alg NPs were synthesized and coated with Eudragit RS100 polymer. For this purpose, FITC-tagged alginate polymer was purchased (medium viscosity, λ_ex_ = 490 nm, λ_em_ = 530 nm) (RuixiBiotech, Changzhou, Jiangsu, China), and Alg and EudAlg NPs were synthesized as mentioned above. Throughout the text, these nanoparticles are designated as F-EudAlg nanoparticles where necessary.

### Loading of EudAlg nanoparticles

For the preparation of loaded EudAlg nanoparticles, a fluorescently labeled peptide was purchased (*M*_w_ = 2.8 kDa, Pepmic, China) and used as a model drug to evaluate encapsulation efficiency and release. For this purpose, the FAM-labeled peptide (1 mg) was dissolved within the sodium alginate polymer solution (1.35 mL of 0.5% w/v), and the protocol mentioned above was followed for the preparation of AlgNPs and EudAlg NPs. The properties of the FAM-labeled peptide are provided in Supporting Information File 2.

### Characterization of nanoparticles

#### Morphology of nanoparticles

The morphological characterization of nanoparticles was done via scanning electron microscopy (SEM, Zeiss, GeminiSEM500) and scanning transmission electron microscopy (STEM, Ziess, GeminiSEM500). For SEM analysis, nanoparticles were air-dried on SEM stabs and coated with gold (4 nm). STEM micrographs were obtained from one drop of nanoparticle suspension dripped on an ultrathin carbon-coated copper grid.

#### Surface composition of nanoparticles

For the analysis of various functional groups on the surface of nanoparticles before and after coating, attenuated total reflectance Fourier-transform infrared spectroscopy (ATR-FTIR) was used. Freeze-dried nanoparticles were directly placed on an ATR crystal, and the infrared spectrum of the samples was obtained in the range of 4000–400 cm^−1^ using a FTIR spectrophotometer (Bruker/Vertex70, USA).

### Particle size and zeta potential distributions

The size of Alg and EudAlg nanoparticles were analyzed by dynamic light scattering (Malvern Zetasizer Nano-ZS) at 25 °C. The nanoparticles were suspended in ddH_2_O; for each sample, three measurements were performed, twelve runs each, to obtain the distributions. Disposable polystyrene cuvettes were used during size distribution analysis, and the distributions were obtained at an angle of 90° with respect to the incident beam. The zeta potential measurements were performed in disposable plain folded capillary zeta potential cuvettes with the same instrument.

### Encapsulation efficiency of EudAlg nanoparticles

To determine the encapsulation efficiency of the model drug (FAM-labeled peptide), into EudAlg NPs, the loaded nanoparticles were prepared as mentioned above. Then, EudAlg nanoparticles (1 mg), which were prepared with an input of 1 mg FAM-labeled peptide, were resuspended in ddH_2_O and fluorescence measurements were carried out (λ_ex_ = 485 nm, λ_em_ = 530 nm) with a black-bottom 96-well plate (BioTek Synergy H1 microplate reader). The amount of encapsulated peptide within the nanoparticles was calculated by using a calibration curve from known amounts of the same FAM-labeled peptide and the corresponding FI values. The encapsulation efficiency was calculated by the following formula:


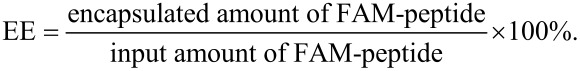


### In situ release of the model drug from EudAlg nanoparticles

The in situ release of the model drug from the nanoparticles was studied as mentioned elsewhere [32,51]. Nanoparticles (2 mg) were resuspended in a freshly prepared dissolution medium (2 mL), that is, either simulated gastric fluid (pH 1.2) or phosphate buffer (pH 6.8), in the absence of enzymes. Alg or EudAlg nanoparticle suspensions were incubated for 7 days with a constant shaking of 50 rpm. At the end of specified time intervals (i.e., after 1, 3, 5, and 7 days), the nanoparticles were pelleted and resuspended again in the corresponding release medium to apply sink conditions. FI values were measured from these nanoparticle suspensions in a black-bottom microplate. The data is presented as “cumulative release vs time”.

### In situ mucoadhesion studies

#### Particle size and zeta potential measurements

To study the mucoadhesion properties of the nanoparticles, mucin (0.5 mg/mL) was mixed with EudAlg nanoparticles at 37 °C for 15 min (1:1, v/v) [52]. The mucoadhesion of the nanoparticles was studied by following the change in particle size and zeta potential distribution (Malvern Zetasizer Nano-ZS).

#### Periodic acid–Schiff stain assay

The colorimetric periodic acid–Schiff stain assay was used to determine the amount of free mucin after the absorption of mucin onto EudAlg nanoparticles [53,59]. For this purpose, first, the periodic acid and Schiff solutions present in the assay kit (BesLab, Türkiye) were used to plot a standard curve from aqueous mucin solutions. Periodic acid solution (200 µL) was added to the mucin samples and incubated in a block heater at 37 °C for 2 h. At the end of incubation, Schiff reagent (200 µL) was added to the solution and incubated for another 30 min at room temperature. Finally, the absorbance of the solution was recorded at 550 nm in a UV spectrophotometer.

To determine the mucoadhesion of the nanoparticles, EudAlg NPs was resuspended in mucin solution (final concentration 0.5 mg/mL, 2 mL for each time interval). The suspensions were incubated at 37 °C while shaking at 50 rpm. After 15 and 60 min of incubation, the nanoparticles were centrifuged (10,000*g* for 10 min), and the absorbance of the supernatants was measured at 550 nm to calculate the amount the free mucin that had not been adsorbed onto the nanoparticles. Nanoparticles resuspended in water (instead of mucin solution) were used as the negative control. The same protocol was applied to pristine mucin solution (0.5 mg/mL), which was used as the reference. The amount of unadsorbed mucin in the supernatants was calculated by using the calibration curve and subtracted from the initial value to obtain the amount of adsorbed mucin onto the nanoparticles. The data is presented as percent of adsorbed mucin with respect to the reference.

#### Interaction of nanoparticles with artificial mucus as a simple model

Diffusion of F-EudAlg nanoparticles through a simple mucus layer was studied based on a similar technique performed by Ungaro and colleagues [61]. Artificial mucus was prepared by dissolving gastric mucin (250 mg), DTPA (0.295 mg), NaCl (250 mg), KCl (110 mg), and sterile egg yolk emulsion (250 µL) in 50 mL ddH_2_O. In another glass vial, gelatin powder was dissolved in hot water (60 °C) to a final concentration of 10% (w/v), and 1 mL of prepared gelatin solution was hardened at room temperature in separate wells of a 24-well plate. The mucin solution (1 mL) was then placed on the hardened gelatin layer, and F-EudAlg nanoparticle suspension (500 µL, 1 mg) was added onto the artificial mucus layer. After 24 h of incubation at room temperature, the mucus layer was discarded, and the gelatin layer was washed and melted. The FI values were recorded from the gelatin layer. As a reference, an aqueous dispersion of F-EudAlg nanoparticles was added on top of the gelatin layer, without mucin, to determine the maximum diffusion of nanoparticles into the gelatin. The data is presented as percent diffusion of nanoparticles with respect to the reference group.

### Determination of cell viability

Cells of the human gastric adenocarcinoma cell line AGS (ATCC CRL-1739) were cultured under normal conditions (37 °C, 5% CO_2_) in RPMI medium, supplemented with 10% FBS, penicillin/streptomycin (1%) and 2 mM ʟ-glutamine. To analyze the cytotoxicity of EudAlg nanoparticles, cells (1 × 10^5^ cells/well) were seeded into 24-well plates and incubated for 1 day for complete attachment. The cells were then treated with EudAlg NPs (1 mg/mL) for 3 days. On days 1, 2, and 3, the medium was withdrawn, and colorless DMEM with 10% alamarBlue was added to each well and incubated for 1.5 h at 37 °C under 5% CO_2_. The optical density was measured at 570 and 595 nm with a microplate reader (BioTek Synergy H1). From these optical density values, percent reduction values (representing the metabolic activity) were calculated for each time point. Data is represented as percent viability, which was calculated by the formula below. The viability percentages of cells at each time point were calculated with respect the corresponding control group.


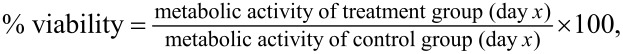


where, day *x* represents day 1, day 2, or day 3 and the control group are cells that were not treated with nanoparticles.

### Internalization of nanoparticles

To analyze the internalization of nanoparticles, 6 × 10^4^ AGS cells were seeded into 12-well plates and treated with F-EudAlg nanoparticles (1 mg/mL). Two incubation intervals were used in the study (1 and 4 h) to study the internalization of nanoparticles in a time-dependent manner. At the end of incubation, the cells were washed twice with PBS and fixed in 4% paraformaldehyde for 15 min at room temperature. The cell nuclei were stained with DAPI (300 mM, 30 min at room temperature), and fluorescence microscopy images were recorded (ZEISS, Axio Observer 3). By using the ZEISS ZEN software, the FITC and DAPI intensity values were obtained from eight different randomly chosen images for each time point, and the ratio FITC intensity/DAPI intensity was used to analyze the internalization of nanoparticles after 4 h.

### In vitro mucus interaction studies

Although AGS cells are known as mucus-secreting gastric epithelial cells [69,78], the presence of mucus secretion from the cells under our culturing conditions were examined with periodic acid–Schiff stain assay in vitro (Supporting Information File 3), which was also used in other studies [73]. For the investigation of interaction between EudAlg nanoparticles and mucus in an in vitro model, AGS cells were used. The development of this assay was inspired by a study performed by Sarparanta and colleagues [79]. AGS cells were seeded on separate 12-well plates at two concentrations (6 × 10^4^ and 1.2 × 10^5^ cells/well) to analyze the increase in mucoadhesion with increasing cell concentrations. Next day, the medium was discarded, and the cells were washed with PBS. The cells in each well were then treated with F-EudAlg nanoparticles (1 mg/mL) for 2 h. At the end of incubation, the nanoparticle solution was removed, and the cells were washed with PBS to remove the nanoparticles not adhered onto the cells. Finally, the cells were detached, centrifuged, and the pellet was resuspended in colorless DMEM. FI intensity measurements (λ_ex_ = 490 nm, λ_em_ = 530 nm) were taken from the pellets containing the cells and possibly adhered nanoparticles. The two control groups of the study were (i) cells without nanoparticle administration (control A) and (ii) empty wells with nanoparticle administration (control B). The relative mucus interaction was calculated through a formula similar to that used in other studies [77]:


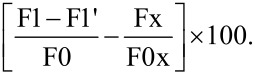


Here, F1 is the fluorescence obtained from the cell pellet treated with NPs after incubation, washing, and centrifugation. F1′ is the fluorescence obtained from the cell pellet of control A. F0 is the fluorescence of nanoparticles administered onto the cells (input). Fx is the fluorescence obtained from control B after incubation, washing, and centrifugation. And F0x is the fluorescence of nanoparticles administered onto the wells (input).

### Statistical analysis

Statistical differences among the study groups in in situ mucoadhesion measurements, PAS stain assay, and internalization studies were assessed by Student’s *t*-tests. The significance threshold was *p* ≤ 0.05. The statistical differences among the groups of viability testing and particle size measurements were assessed by one-way ANOVA with Tukey post-hoc analysis. A *p* value ≤ 0.05 was considered statistically significant.

## Supporting Information

File 1 Coating of alginate nanoparticles by Eudragit RS100 polymer.

File 2 Properties of FAM-labeled peptide.

File 3 Periodic acid–Schiff staining of cells.

## Data Availability

All data that supports the findings of this study is available in the published article and/or the supporting information of this article.
