# Supplementary material for: Development of a mucoadhesive drug delivery system and its interaction with gastric cells
Source: Beilstein J Nanotechnol. 2025 Mar 13;16:371–84. doi: 10.3762/bjnano.16.28 (PMC11912645; doi:10.3762/bjnano.16.28)
Supplement: File 1 — Coating of alginate nanoparticles by Eudragit RS100 polymer. [file Beilstein_J_Nanotechnol-16-371-s001.pdf]

BEILSTEIN JOURNAL OF NANOTECHNOLOGY

Supporting Information

for

Development of a mucoadhesive drug delivery system and its interaction with gastric cells

Ahmet Baki Sahin, Serdar Karakurt and Deniz Sezlev Bilecen

Beilstein J. Nanotechnol. **2025,** *16,* 371–384. doi:10.3762/bjnano.16.28

Coating of alginate nanoparticles by Eudragit RS100 polymer

License and Terms: This is a supporting information file under the terms of the Creative Commons Attribution License (https://creativecommons.org/licenses/by/4.0). Please note that the reuse, redistribution and reproduction in particular requires that the author(s) and source are credited and that individual graphics may be subject to special legal provisions.
The license is subject to the *Beilstein Journal of Nanotechnology* terms and conditions: (https://www.beilstein-journals.org/bjnano/terms)

Coating of alginate nanoparticles with Eudragit RS100 polymer

To study the interaction of Eudragit RS100 polymer with alginate nanoparticles during coating, Eudragit RS100 polymer was labeled with rhodamine B isothiocyanate (Rh-Eud polymer; λ_{ex} = 540 nm, λ_{em} = 575 nm). To do this, Eudragit RS30D suspension (1 mL) was mixed with 20 µL rhodamine B isothiocyanate solution (10 mg/mL, Sigma, 283924) and incubated at room temperature on a magnetic stirrer (200 rpm, 3 h). At the end of incubation, the suspension was centrifuged (10000*g*, 10 min) and washed six times to remove the unbound rhodamine B. Then, alginate nanoparticles were prepared by using FITC-tagged alginate polymer following the procedure mentioned above to form F-Alg nanoparticles. Finally, the Rh-Eud polymer was used to coat F-Alg nanoparticles and Rh-Eud-F-Alg nanoparticles were synthesized. The interaction between Eudragit RS100 polymer and alginate nanoparticles were studied by measuring the fluorescence spectrum of particles in the range of 515–595 nm using the excitation wavelength of F-Alg (λ_{ex} = 490 nm) (Figure S1). The emission spectrum of Rh-Eud polymer (λ_{ex} = 530 nm) is provided in Figure S2.

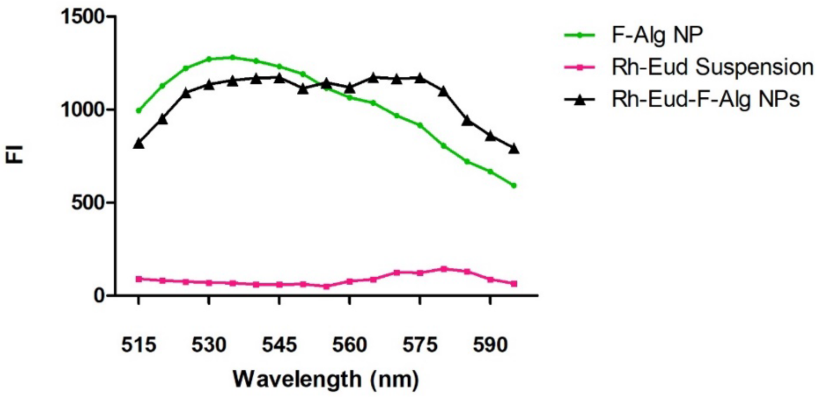

Figure S1: Fluorescence spectra of F-Alg NP, Rh-Eud suspension, and Rh-Eud-F-Alg NPs. A secondary peak was observed in the spectrum of Rh-Eud-F-Alg nanoparticles with λ_{ex} = 490 nm, which may indicate the close interaction of Rh-Eud with F-Alg nanoparticles.

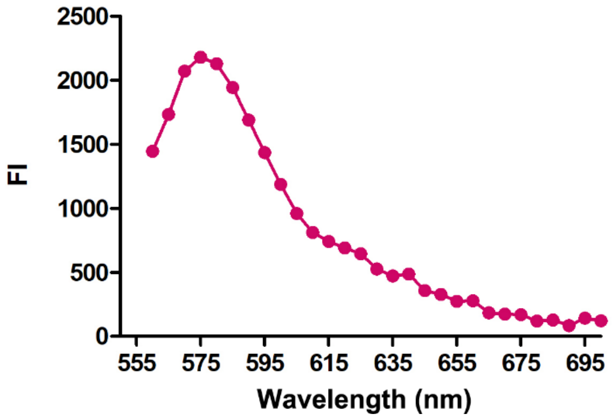

Figure S2: Fluorescence spectrum of Rh-Eud suspension with λ_{ex} = 530 nm. This supports that the emission obtained from F-Alg nanoparticles at 530 nm can excite the Rh-Eud polymer. The excitation of Rh-Eud polymer with 530 nm results in emission with λ_{em} at 575 nm.
