# Supplementary material for: Development of a mucoadhesive drug delivery system and its interaction with gastric cells
Source: Beilstein J Nanotechnol. 2025 Mar 13;16:371–84. doi: 10.3762/bjnano.16.28 (PMC11912645; doi:10.3762/bjnano.16.28)
Supplement: File 2 — Properties of FAM-labeled peptide. [file Beilstein_J_Nanotechnol-16-371-s002.pdf]

BEILSTEIN JOURNAL OF NANOTECHNOLOGY

Supporting Information

for

Development of a mucoadhesive drug delivery system and its interaction with gastric cells

Ahmet Baki Sahin, Serdar Karakurt and Deniz Sezlev Bilecen

Beilstein J. Nanotechnol. **2025,** *16,* 371–384. doi:10.3762/bjnano.16.28

Properties of FAM-labeled peptide

License and Terms: This is a supporting information file under the terms of the Creative Commons Attribution License (https://creativecommons.org/licenses/by/4.0). Please note that the reuse, redistribution and reproduction in particular requires that the author(s) and source are credited and that individual graphics may be subject to special legal provisions.
The license is subject to the *Beilstein Journal of Nanotechnology* terms and conditions: (https://www.beilstein-journals.org/bjnano/terms)

Properties of the FAM-labeled peptide

The mass spectrum of the FAM-labeled peptide that was used as a model drug molecule is provided in Figure S1. The emission spectrum (λ_{ex} = 485 nm) of the purchased FAM-labeled peptide is given in Figure S2.

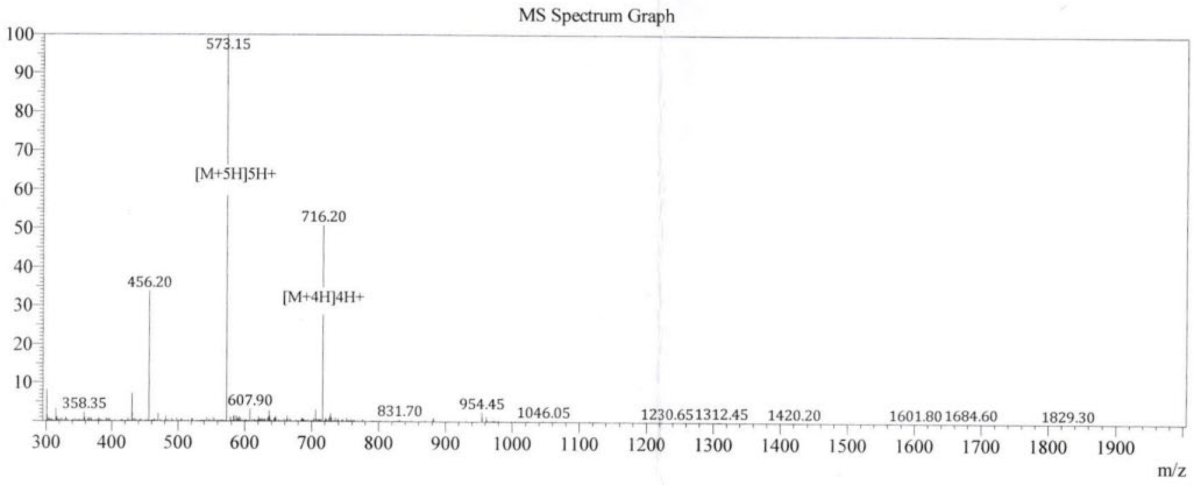

Figure S1: Mass spectrum of the FAM-labeled peptide (sequence: lauric acid–VVAGFSGGNCRGFRRRCFCT{K(5-FAM)}–NH_2). The mass spectrum result of the model peptide is $[M + 4H]^{4+}_{calc}$ = 716.34, $[M + 4H]^{4+}_{obs}$ = 716.20; $[M + 5H]^{5+}_{calc}$ = 573.07, $[M + 5H]^{5+}_{obs}$ = 573.15.

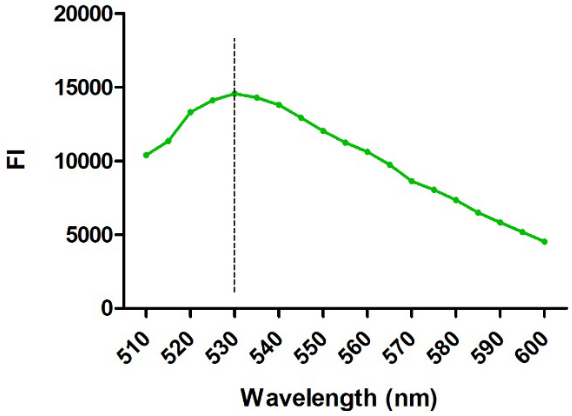

Figure S2: Emission spectrum of the FAM-labeled peptide, λ_{ex} = 485 nm.
