# Supplementary material for: Development of a mucoadhesive drug delivery system and its interaction with gastric cells
Source: Beilstein J Nanotechnol. 2025 Mar 13;16:371–84. doi: 10.3762/bjnano.16.28 (PMC11912645; doi:10.3762/bjnano.16.28)
Supplement: File 3 — Periodic acid–Schiff staining of cells. [file Beilstein_J_Nanotechnol-16-371-s003.pdf]

BEILSTEIN JOURNAL OF NANOTECHNOLOGY

Supporting Information

for

Development of a mucoadhesive drug delivery system and its interaction with gastric cells

Ahmet Baki Sahin, Serdar Karakurt and Deniz Sezlev Bilecen

Beilstein J. Nanotechnol. **2025,** *16,* 371–384. doi:10.3762/bjnano.16.28

Periodic acid–Schiff staining of cells

License and Terms: This is a supporting information file under the terms of the Creative Commons Attribution License (https://creativecommons.org/licenses/by/4.0). Please note that the reuse, redistribution and reproduction in particular requires that the author(s) and source are credited and that individual graphics may be subject to special legal provisions.
The license is subject to the *Beilstein Journal of Nanotechnology* terms and conditions: (https://www.beilstein-journals.org/bjnano/terms)

Periodic acid–Schiff (PAS) staining of cells

6 × 10^4 cells were seeded on 24-well plates and incubated for two days, then fixed with 4% paraformaldehyde for 20 min. After washing with PBS, the cells were incubated with periodic acid (0.5% for 10 min), washed with PBS, and incubated with Schiff's reagent for 30 min. The cells were then washed with water, and images were taken under an inverted light microscope. The cells with purple-red-stained cytoplasm were counted as mucus-positive cells (Figure S1).

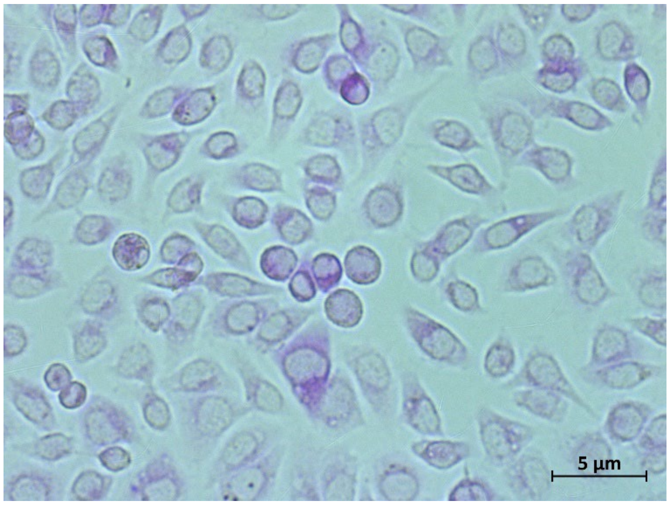

Figure S1: Light microscopy image of mucus-positive AGS cells.
